# Design and Parametric Study of the Magnetic Sensor for Position Detection in Linear Motor Based on Nonlinear Parametric Model Order Reduction

**DOI:** 10.3390/s17071543

**Published:** 2017-07-01

**Authors:** Sarbajit Paul, Junghwan Chang

**Affiliations:** Mechatronics System Research Laboratory, Department of Electrical Engineering, Dong-A University, Busan 49315, Korea; spaul@donga.ac.kr

**Keywords:** dynamic mode decomposition, linear motor, magnetic sensor, model order reduction, multipolar moment matching method, parameterization, proper orthogonal decomposition

## Abstract

This paper presents a design approach for a magnetic sensor module to detect mover position using the proper orthogonal decomposition-dynamic mode decomposition (POD-DMD)-based nonlinear parametric model order reduction (PMOR). The parameterization of the sensor module is achieved by using the multipolar moment matching method. Several geometric variables of the sensor module are considered while developing the parametric study. The operation of the sensor module is based on the principle of the airgap flux density distribution detection by the Hall Effect IC. Therefore, the design objective is to achieve a peak flux density (PFD) greater than 0.1 T and total harmonic distortion (THD) less than 3%. To fulfill the constraint conditions, the specifications for the sensor module is achieved by using POD-DMD based reduced model. The POD-DMD based reduced model provides a platform to analyze the high number of design models very fast, with less computational burden. Finally, with the final specifications, the experimental prototype is designed and tested. Two different modes, 90° and 120° modes respectively are used to obtain the position information of the linear motor mover. The position information thus obtained are compared with that of the linear scale data, used as a reference signal. The position information obtained using the 120° mode has a standard deviation of 0.10 mm from the reference linear scale signal, whereas the 90° mode position signal shows a deviation of 0.23 mm from the reference. The deviation in the output arises due to the mechanical tolerances introduced into the specification during the manufacturing process. This provides a scope for coupling the reliability based design optimization in the design process as a future extension.

## 1. Introduction

To precisely control the mover in a linear motor, the correct information about the mover position is needed. There are various methods available to detect the mover position using optical magnetic and capacitive sensors. Among the optical sensors, optical encoders are widely used to detect the mover position. There are different kinds of optical encoders available for this purpose, but for a linear motor application, considering the linear movements, installation of linear optical encoders would be expensive. Optical sensors can be substituted by capacitive type sensors, but again, their complex design and working principle could be constraints in the application to linear motors. A cost effective and accurate substitute could be a linear position magnetic sensor. There are different kinds of magnetic sensors available in the literature [1,2,3,4,5,6]. Previously, authors have proposed a Hall Effect-based linear magnetic sensor module which can accurately detect the mover position of linear motors [7].

In [7], the authors applied the finite element method (FEM) to analyze the flux density profile of a linear position magnetic sensor module. FEM is considered as a useful and powerful numerical tool to analyze magnetostatic and magnetodynamic systems. From the manufacturing point of view, designing a magnetic sensor includes a parametric study, optimization and advanced studies based on manufacturing tolerance to achieve the desired output from the sensor module. All these above-mentioned operations require multiple FEM studies of the basic model. Under this condition, FEM analysis of the magnetic sensor module becomes expensive in terms of storage space, numerical computation and simulation time.

To overcome this problem associated with the FEM, model order reduction (MOR)-based methods are extensively used. Initially, MOR was introduced in the field of structural and fluid dynamics [8]. Different types of MOR methods have been documented in the literature over the last two decades. Works based on the proper orthogonal decomposition (POD) method are ubiquitous for dimensionality reduction of different engineering systems [9,10,11,12,13,14].

In magnetic field problems, the application of the POD-based MOR is relatively new. The use of POD-based MOR to analyze magnetostatic and magnetodynamic problems is documented in [10,15,16]. Hanneron et al. presented their numerical study of an electrical motor using POD in [17]. Also, Far et al. introduced a multiple input case for an electrical machine in [18]. To incorporate the nonlinearity in the electromagnetic systems, references [19,20] presented the use of the so-called Empirical Interpolation Method (EIM) and Discrete Empirical Interpolation Method (DEIM) along with POD. These methods give a good approximation, but DEIM is a numerically complicated method. An alternative to the POD-DEIM, dynamic mode decomposition (DMD), can be considered. DMD is used to approximate the nonlinearity associated with the problem statement. The special feature of the DMD, which allows the decomposition of data into spatiotemporal modes as well as associates the data to temporal Fourier modes, makes it a suitable candidate to approximate the nonlinearity. A detailed study of the DMD and its applications was presented by Tu et al. [21].

To analyze the magnetic sensor module for a linear motor mover position detection problem, in this paper, a POD-DMD-based nonlinear magnetic sensor module is considered. To the best of our knowledge, the implementation of the POD-DMD-based MOR on a magnetic system has not been addressed in the literature before. The designed framework based on the reduced model provides a computationally efficient and time saving method for the parameterization and optimization process of the sensor module compared with the traditional FEM approach. For the special kind of the optimization processes which consider manufacturing tolerances, the proposed framework can provide a collective gain in terms of noncompliance cost as well as computational speed, therefore, makes the proposed method invaluable for the manufacturers.

The paper is organized as follows: Section 2 introduces the basic mathematical framework of the MOR. The implementation of the POD-DMD-based MOR on a nonlinear partial differential equation and its comparison with other existing nonlinear MOR methods is presented in Section 3. Section 4 discusses the linear position magnetic sensor for linear motor position detection with the POD-DMD-based MOR of the sensor module and the parametric study. In Section 5, experiments using the manufactured sensor module are presented. The paper concludes in Section 6 with a discussion of the possible future extensions of this work.

## 2. A Formal MOR Framework for Nonlinear System

To develop a computationally inexpensive model of the magnetic sensor module for a linear motor, using MOR, the required mathematical framework is discussed below.

### 2.1. Nonlinear System Model

A nonlinear system can be formulated as an ordinary differential equation (ODE) of the form:

(1) $$\frac{d}{dt}y(t)=Ay(t)+F(y(t))$$

where, *t* ∈[0,T] is the time, **y**(*t*) = ${\left[y_{1}\left(t\right),y_{2}\left(t\right)\ldots ,y_{n}\left(t\right)\right]}^{T}\in {\mathbb{R}}^{n\times n}$ is a state vector, **F** is a nonlinear function of **y**(*t*), F = ${\left[F(y_{1}\left(t\right)),F(y_{2}\left(t\right))\ldots ,F(y_{n}\left(t\right))\right]}^{T}$ and **A** $\in {\mathbb{R}}^{n\times n}$ is a given matrix.

This general description of the nonlinear system arises in many real life systems. In such cases, it is difficult and time-consuming to analyze the very large *n*-dimensional system. Thus a computationally effective method based on MOR can be an alternative approach to the previously developed FEM method.

### 2.2. Numerical Concept of MOR Using POD

One of the popular MOR methods is POD, which is a projection-based MOR method. The idea of POD was first proposed by Sirovich [11]. This method is same as the Singular Value Decomposition (SVD) in a finite dimensional space or in Euclidean space. POD can efficiently extract the basis elements that contain the characteristics of the targeted system. Basis elements known as POD basis can be found using the method of snapshot [13]. To obtain the snapshots, let us assume that the system is solved for *m* exclusive times and the data is arranged in a snapshot matrix as **S** $=\left[y^{1},y^{2},\ldots ,y^{m}\right]\in {\mathbb{R}}^{n\times m}$ with a rank *r*. Then, SVD is applied on **S** as follows:

(2) $$S=U\Sigma V^{*}$$

where, $U=\left[u_{1},u_{2},\ldots ,u_{r}\right]\in {\mathbb{R}}^{n\times r}$ and $V=\left[v_{1},v_{2},\ldots ,v_{r}\right]\in {\mathbb{R}}^{m\times r}$ are the orthogonal matrices and $\Sigma =\mathrm{diag}\left(\sigma_{1},\sigma_{2},\ldots ,\sigma_{r}\right)\in {\mathbb{R}}^{r\times r}$ is a diagonal matrix, with $\sigma_{1}\geq \sigma_{2}\geq \ldots \geq \sigma_{r}\geq 0$, known as the singular values. The rank of **S** is r≤min(n,m).

Using energy distribution of the singular value spectrum $\sigma_{1},\sigma_{2},\ldots ,\sigma_{r},$ the state vector **y**(*t*) in Equation (1) can be approximated to a reduced basis by a vector $y_{k}\left(t\right)\in {\mathbb{R}}^{n\times k}$ with k≤n as follows:

(3) $$y(t)=\Psi y_{k}(t)$$

where, is Ψ called as the projection operator. The expression for Ψ can be obtained from Equation (2) by considering the energy distribution of $\sigma_{1},\sigma_{2},\ldots ,\sigma_{r}.$ Then using Ψ, the reduced model of Equation (1) can be written as follows:

(4) $$\frac{d}{dt}y_{k}(t)=\Psi^{T}A\Psi y_{k}(t)+\Psi^{T}F(\Psi y_{k}(t))$$

From Equation (4) it can be noticed that POD can provide an excellent reduced model of Equation (1) order k≤n. However, the dimensionality reduction is usually limited to the linear terms. POD alone cannot provide an appropriately reduced model when nonlinearity is present. This is evident from Equation (4) because to compute the nonlinearity term in Equation (4), $y_{k}\left(t\right)\in {\mathbb{R}}^{k}$ needs to be expanded to an *n* dimensional vector $\Psi y_{k}\left(t\right)\in {\mathbb{R}}^{n}$. Thereafter the nonlinearity $F\left(\Psi y_{k}\left(t\right)\right)$ is calculated and returned back to the reduced model. Thus, the approximation of the nonlinearity is computationally expensive since the high dimensional full model needs to be evaluated to calculate the nonlinearity.

To overcome this difficulty, methods such as EIM and DEIM were implemented along with POD [19,20]. Magnetostatic and magnetodynamics nonlinear systems were analyzed using the POD-DIEM method previously. Although POD-DEIM is computationally efficient in magnetic device design, it is a complicated method to implement. For our design process, an alternative to the POD-DEIM method, we used recently developed DMD in this paper. DMD is an equation-free spatiotemporal approach. Thus the analysis of the highly nonlinear system can simply be done compared with the POD-DIEM method. To the best of our knowledge, the implementation of DMD to achieve the reduced model of a magnetic device has not yet been documented in the literature.

## 3. Application of the DMD and Its Comparison with the Other Nonlinear MOR Methods

### 3.1. Basic Idea of DMD 

The very initial study of DMD dates back to 1931, when Bernard Koopman introduced Koopman operator. Thereafter in 2008, Schmid developed the DMD-based model for nonlinear complex flow to extract the dynamic flow data [22]. There is a good number of documented literature which established the connectivity between Koopman operator and DMD [23].

Let us consider a set of snapshot matrix **S** $=\left[y^{1},y^{2},\ldots ,y^{m}\right]\in {\mathbb{R}}^{n\times m}$. If we assume that the data are generated by the linear dynamics as shown below:

(5) $$y_{k+1}=A_{y}y_{k}$$

where, $A_{y}$ is some unknown matrix which approximates the dynamics of a nonlinear dynamical system. Thus to find the system behavior, our aim is find **A_y_**. It can be done using the DMD as shown in Algorithm 1 [21],
**Algorithm 1** SVD based.**1** Arrange the data of snapshot matrix **S** into two different matrices, as:
$$S^{\prime }≜\left[y^{1}\ldots y^{m-1}\right];S^{\prime \prime }≜\left[y^{2}\ldots y^{m}\right]$$ **2** Compute the SVD of $S^{\prime }$; $S^{\prime }=U\Sigma V^{*}$ where, $U=\left[u_{1},u_{2},\ldots ,u_{r}\right]\in {\mathbb{R}}^{n\times r}$ and $V=\left[v_{1},v_{2},\ldots ,v_{r}\right]\in {\mathbb{R}}^{m\times r}$ are orthogonal matrices and $\Sigma =\mathrm{diag}\left(\sigma_{1},\sigma_{2},\ldots ,\sigma_{r}\right)\in {\mathbb{R}}^{r\times r}$ is a diagonal matrix. **3** Define a matrix $\tilde{A_{y}}$ such that, $\tilde{A_{y}}≜U^{*}\Sigma^{-1}VS^{\prime \prime }$; here, $\tilde{A_{y}}$ is the k×k projection of $A_{y}$ such that, $\tilde{A_{y}}$ = $U^{*}A_{y}U$. **4** Compute the eigenvalues and eigenvectors of $\tilde{A_{y}}$ as: $\tilde{A_{y}}w=\lambda w$. **5** Calculate the DMD mode corresponding to *λ*: $\Psi^{\mathrm{DMD}}=S^{\prime \prime }V\Sigma^{-1}w$.

### 3.2. DMD Approach for Nonlinear System Model

To begin with, let us find the snapshot matrix ${\left\{y^{m}\right\}}_{i=1}^{m}$ of the linear section of Equation (1) for a given time ${\left\{t_{i}\right\}}_{i=1}^{m}$ and compute the POD basis $\Psi^{\mathrm{POD}}$ of rank *k*. For the nonlinear section, we need to collect the snapshot for the nonlinearity, ${\left\{F\left(t_{i},y^{m}\right)\right\}}_{i=1}^{m}$ and apply the Algorithm 1 mentioned above. Thus DMD approximation of the nonlinear section can be written as follows:

(6) $$F\left(t,y\right)=\overset{k}{\underset{i=1}{\sum }}p_{i}\psi_{i}^{\mathrm{DMD}}\exp\left(w_{i}t\right)$$

where, $\psi_{i}^{\mathrm{DMD}}$ are the DMD basis for rank *l* for the nonlinear section F(t,y) of Equation (1), $p_{i}$ is the initial condition and $w_{i}$ are the eigenvalues of $\tilde{A_{y}}$ mentioned in Algorithm 1. By applying the compact notation to Equation (6) and substituting it to Equation (1) along with the approximation of the linear section, we obtain the reduced model of the whole system as follows:

(7) $$\frac{d}{dt}y_{k}(t)=A^{k}y_{k}(t)+\Psi^{T}\Psi^{\mathrm{DMD}}\mathrm{diag}\left(e^{w^{\mathrm{DMD}}t}\right)p;\;p={\left(\Psi^{\mathrm{DMD}}\right)}^{†}F_{1}\in {\mathbb{R}}^{l}$$

where, † denotes the Moore-Penrose pseudoinverse. Now, if we compare the dimensions of Equation (7) with those of Equation (4), the dimension of $A^{k}$ is same in both Equations (4) and (7). The dimension of $\Psi^{T}\Psi^{\mathrm{DMD}}$ in Equation (7) is ${\mathbb{R}}^{l\times r}$ and is less than the dimension of the full model. Thus the POD-DMD model has a dimension which does not depend on the dimension of the full model. This method also provides a speed up in the nonlinearity analysis compared with the DEIM method, because using this method there is no need to compute the nonlinearity further in offline.

### 3.3. Numerical Example

**Figure 1:**
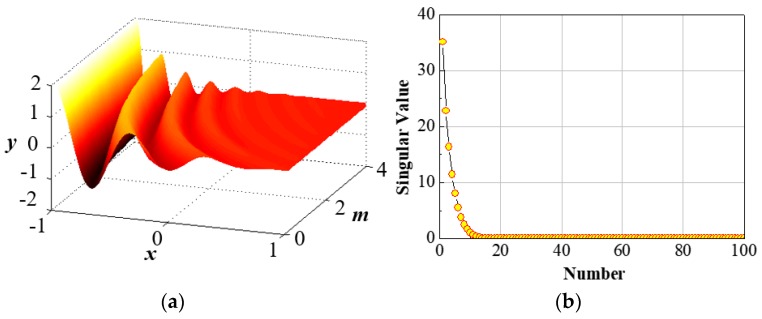
(**a**) Surface plot showing the distribution of **y**; (**b**) Singular value energy distribution by SVD.

**Figure 2:**
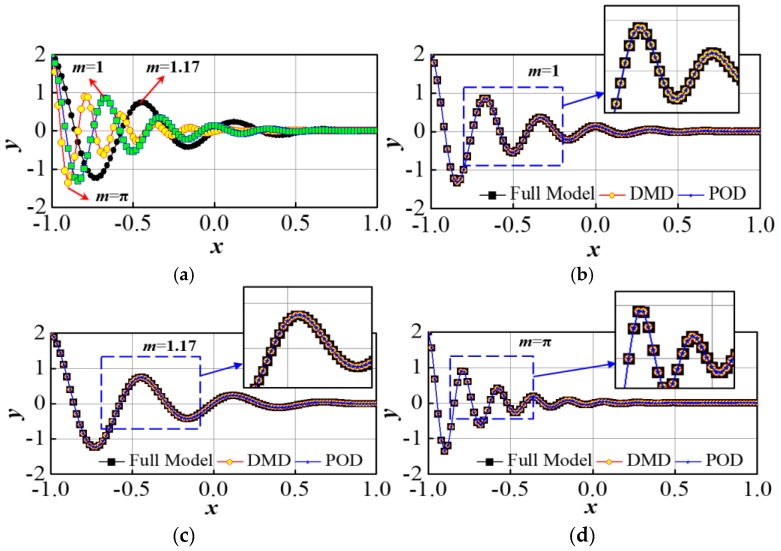
(**a**) Distribution of y for *m* = 1, 1.17 and π with full model analysis; Comparison of the DMD based reduced model with full model and DEIM model for (**b**) *m* = 1, (**c**) *m* = 1.17 and (**d**) *m* = π.

To check the validity of the mathematical proposition in the above sections, a numerical example of the following parameterized function is considered as follows [24]:

(8) $$y\left(x;m\right)=\left(1-x\right)\cos\left(3\pi m\left(x+1\right)\right)e^{-\left(1+x\right)m}$$

where, y:Ω×D↦ℝ,x∈[−1,1] and m∈D=[1,π]. Let ${\left[{\left\{x_{i}\right\}}_{i=1}^{n}\right]}^{T}\in {\mathbb{R}}^{n}$, with *n* = 100. We also define $f:D\mapsto {\mathbb{R}}^{n}$ by $f={\left\{y\left(x_{i};m\right)\right\}}_{i=1}^{n}\in {\mathbb{R}}^{n}$. Figure 1a shows the behavior of the function (8) with the variation of *x* and *m* within the predefined limits. The energy distribution of the singular values is presented in Figure 1b. As shown in Figure 1b, the first 10 modes have the highest singular values. Therefore, they constitute most of the system energy and thus, rather using *n* = 100, the whole system can be approximated using only *n* = 10 modes. This shows the requirement of the Model order reduction to address this issue associated with high dimensional system. Now, the effectiveness of DMD in approximating the full model for Equation (8) is checked by considering three values of *m* namely, m={1,1.17,π}. Figure 2a shows the full model simulation for three different values of *m* respectively. In Figure 2b–d, the approximation of **y** (*m* = 1), **y** (*m* = 1.17), and **y** (*m* = 3.14) using DMD is compared with the full model and DEIM. As shown in above, DMD is capable of reconstructing the full model without significant deviation and it has the potential to substitute DEIM based nonlinear system approximation. Thus, DMD combined with POD can be used to design the desired sensor module for the linear motor position detection.

## 4. A Linear Position Magnetic Sensor Module for Linear Motor Mover Position Detection

### 4.1. Magnetic Sensor Module and Geometric Parameters associated with the model

**Figure 3:**
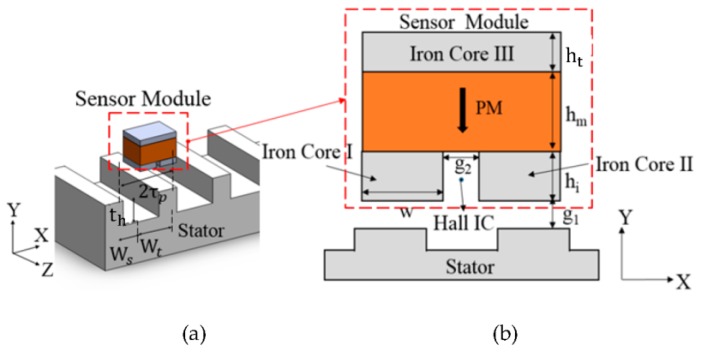
Linear position sensor module with the stator: (**a**) Sensor module arrangement over stator; (**b**) sensor module with different parameters.

The three-dimensional view of the linear position magnetic sensor and its positioning over the stator of the linear motor on XYZ plane is shown in Figure 3a [7].

As shown in Figure 3b, the magnetic sensor module consists of a permanent magnet (PM), made of Nd-Fe-B and three other iron steel cores I, II and III respectively. In between the iron cores I and II, a Hall IC is placed. The Hall IC is A1324 linear Hall Effect sensor manufactured by Allegro Microsystems LLC (Worcester, MA, USA). The PM is magnetized along negative Y direction and the desired magnetic flux density is measured along X direction (B_X_) at the center point of airgap $g_{2}$ in between the iron core I and II. The iron cores I, II and III are used to provide low reluctance path for the magnetic flux.

**Figure 4:**
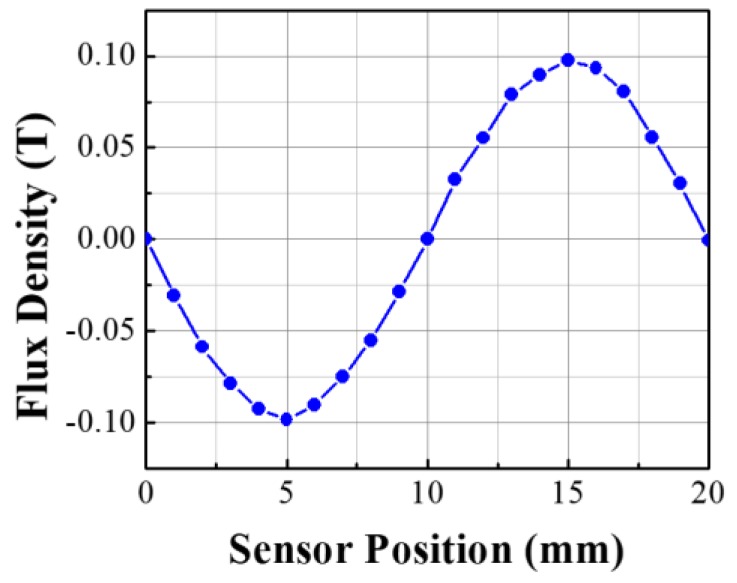
Distribution of the flux density at the center of $g_{2}$ for two pole pitch movement of the sensor module over the stator tooth-slot.

The working principle of the linear position sensor module is based on the detection of the flux density variation with the variation of the reluctance of the flux path. Due to the presence of the alternate tooth-slot structure of the linear motor stator, at the position wherein the mover module is aligned with the stator teeth or placed in between two stator teeth, due to symmetry, flux density B_x_ will be zero. Thus for two pole pitch displacement, the flux density distribution at the central point of $g_{2}$ will be a sinusoid as shown in Figure 4.

To obtain a minimum required output of 1 V peak to peak of the Hall IC, the required peak flux density (PFD) for the sensor module should be at least 0.1 T. Added to that, the total harmonic distortion (THD) is expected to be less than 3%. To achieve this goal, sensor module needs to undergo a detail parametric study. To carry out the parametric study, instead of using traditional FEM, the POD-DMD based reduced model is designed based on the principle mentioned in Section 3, to achieve faster and computationally efficient parametric model.

As shown in Figure 3b, to do the parametric analysis $g_{1},g_{2},h_{i},h_{t},\;\mathrm{and}\;w$ are considered as the variables. The linear motor configuration and sensor variable ranges for the parametric design are listed in Table 1.

### 4.2. Parametric Design Using the Reduced Model Based on POD-DMD

Prior to carrying out the parametric study, the information of the design parameters and their variations are required. The behavior of the parameter variation is modeled using the multiparameter moment matching method-based parametric model order reduction (PMOR). The overall computational operation of the magnetic sensor design can be divided into: (1) initial parameter model based on FEM, (2) parameterization based on multiparameter moment matching method and (3) POD-DMD-based MOR.

#### 4.2.1. Multi-Parameter Moment Matching Method Based PMOR for Linear Magnetic System

The behavior of the magnetic sensor can be studied by using magnetic vector potential. For a magnetic device, the device can be described by the magnetostatic problem of Maxwell’s equation. On any domain D, Maxwell’s equation for a magnetic system can be defined by the equations:

(9) $$\mathrm{curl}\;H=J_{m}$$

(10) div B=0

where, **H** is the magnetic field intensity, **B** is the magnetic flux density and **J**_m_ is the current density due to the PM equivalent current source. Now according to the constitutive law, we can write:

(11) $$H=\upsilon \left[B-B_{r}\right]$$

where, υ is the magnetic reluctivity and $B_{r}$ is the remanent magnetic flux density. Maxwell’s Equations (9) and (10) can be solved by vector potential finite element formulation. Magnetic flux density can be expressed in vector potential as, curl A=B. Putting this value into the Equation (9) and using Equation (11), we can write:

(12) $$\mathrm{curl}\left[\upsilon \;\mathrm{curl}\;A\right]=\mathrm{curl}\;\left[\upsilon \;B_{r}\right]+J_{m}$$

Using the Galerkin Method, Equation (12) can be formulated as:

(13) M U=F

where, $M_{m\times m}$ is the stiffness matrix, $U_{m\times 1}$ is the vector of the nodal potentials with m degrees of freedom(DoF), $F_{m\times 1}$ is the source vector. Now, let us consider some design parameters $p=\left(p_{1},p_{2},\cdots p_{np}\right)$ for the sensor module which varies during the design process. Equation (13) can be modified as:

(14) M(p)U(p)=F(p)

From Equation (14), the vector potential is a function of **p**. To analyze the system under parameter variation an easy way is to remesh each geometry corresponding to new parameter set **p**. A new remeashing of the domain of consideration adds a numerical noise on the output data, because the mesh changes from one parameter set to another. Also, due to the change of shape function extra computation is needed. To avoid such a computational burden, different methods to incorporate the parameter variation effects are studied [25,26]. In the proposed framework, the effects of parameters are included into the system using the multiparameter moment based method [25].

To reduce the computational time required to solve the parameterized system (14), the POD method is applied [2] and the reduced model can be written as:

(15) $$\Psi^{T}M(p)\Psi U_{r}(p)=\Psi^{T}F(p)$$

(16) $$U(p)\approx \Psi U_{r}(p)$$

where, **Ψ** is the discrete projection operator, calculated by the method of snapshot and $U_{r}$ is the approximation of **U** in Equation (14) with the POD method presented in Section 2.2. The DoF of the reduced model in Equation (12) is *r* (*r* << *m*).

#### 4.2.2. POD-DMD based PMOR

In the real world, no magnetic system is linear. Thus, to design the magnetic sensor module accurately, the nonlinear dependency and magnetic saturation must be considered. For an instance, for the nonlinear magnetic device, υ in Equations (11) and (12) varies nonlinearly. As mentioned in Section 2.2, for the reduced model in Equation (15), calculation of **M** and **F** will be computationally expensive. To achieve the reduced model of **M** and **F**, along with the POD model of Equation (15), we applied the DMD method mentioned in Section 3. By considering the Equation (7), Equation (15) can be modified as follows:

(17) $$M_{r}(p)U_{r}(p)=F_{r}(p)$$

where:

$$M_{r}(p){=\Psi }^{\mathrm{DMD}}\mathrm{diag}\left(e^{w^{\mathrm{DMD}}t}\right)\times \Psi^{T}M(p)\Psi$$

(18) $$F_{r}(p)=\Psi^{\mathrm{DMD}}\mathrm{diag}\left(e^{w^{\mathrm{DMD}}t}\right)\times \Psi^{T}F$$

The combination of POD and DMD is attractive in terms of computational cost, but during the process, we need to consider the error related to the approximation. A proper selection of POD and DMD modes can reduce the approximation error of the system.

### 4.3. Parametric Design and Result

#### 4.3.1. Parametric Modeling for 2D Model

**Figure 5:**
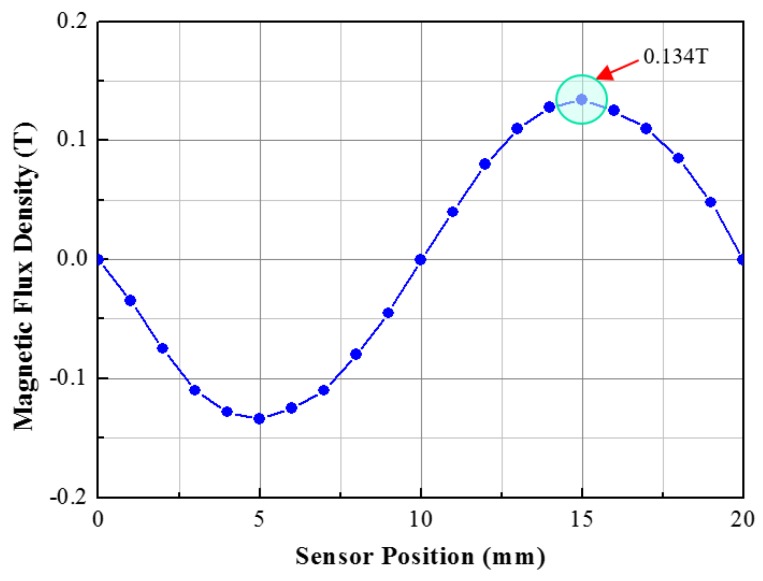
Distribution of flux density with the 2D initial model of the sensor module.

**Figure 6:**
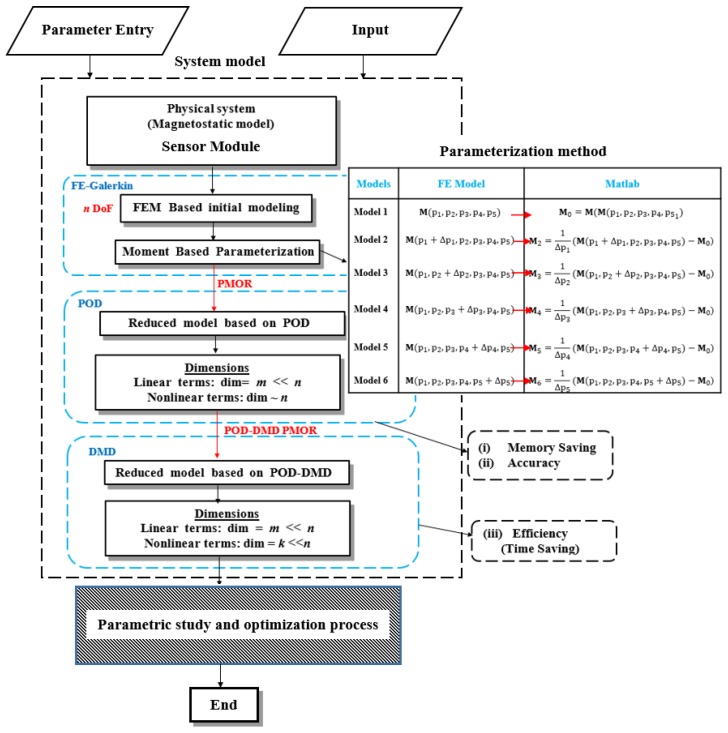
Flowchart for System Design and Analysis.

**Figure 7:**
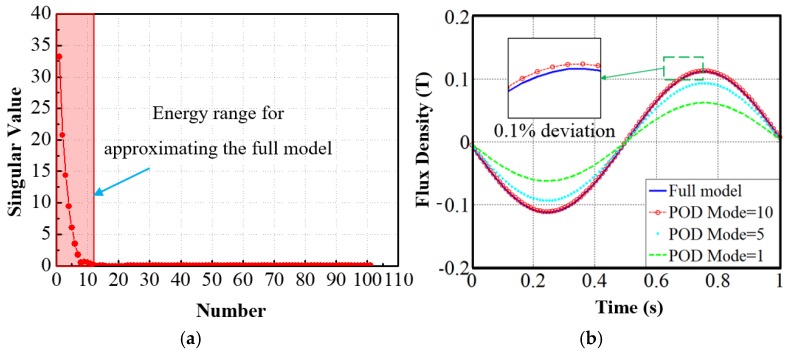
(**a**) Singular value spectrum of the snapshot matrix and (**b**) flux density distribution plot for the full model and reduced models with different POD modes.

To start with, one initial FE model of the sensor module, which is bit arbitrary is modeled using Flux 2D software to investigate the performance. The five design variables mentioned in Table 1 are chosen as: $\left(g_{1},g_{2},h_{i},h_{t},w\right)$ = (1, 2, 3, 0, 7). Under these variables, the flux density variation at the center of the sensor module of Figure 3b is shown in Figure 5, where the PFD is 0.134 T and the THD is 3.18%. The flux density thus obtained fulfills the requirement of minimum PFD for our sensor module. However, the THD needs to be minimized to reach under 3% as per requirement. To obtain a parameter set that satisfies the design requirements, the effect of each individual parameters and their combined effects on PFD and THD needs to be analyzed. The whole process is carried out as shown in the flowchart in Figure 6. As shown in Figure 6, after selecting the five geometric parameters in our case, 6 traditional FE models for the sensors are studied to list their effects on the sensor performance. It is shown in the “Parameterization Method” of the flowchart. The values for the “Parameterized Method” table shown in flowchart are found with the FE model data. Then, they are linked to the reduced model framework in MATLAB platform. Based on the singular value spectrum of the full FE model data in Figure 7a, three different choices of POD modes are made, as shown in Figure 7b. With the increase of the POD modes, the reduced model can be approximated with less deviation from the full model. The choice of the POD modes lies on the user by considering the level of accuracy and computational efficiency. For the optimization process of the sensor module, 10 POD modes are considered, which gives only 0.1% deviation from the full model in terms of the flux density waveform, showing excellent accuracy.

**Figure 8:**
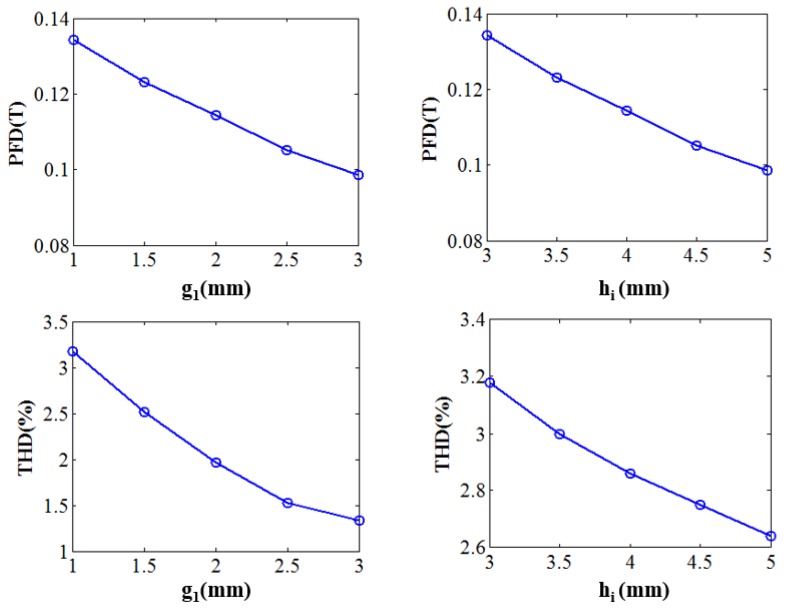

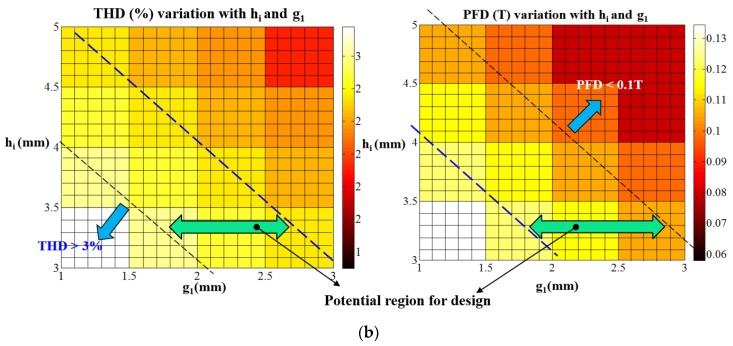
(**a**) variation of PFD and THD with $g_{1}{\;\mathrm{and}\;h}_{i}$ variation (**b**) design compromise between $g_{1}{\;\mathrm{and}\;h}_{i}$ for PFD and THD.

**Figure 9:**
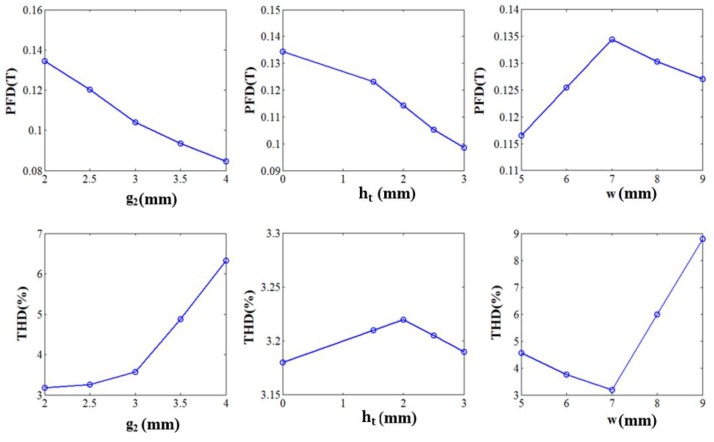
PFD and THD variation with the variation of $g_{2},h_{t},\;\mathrm{and}\;w.$

Using the POD-DMD model of the sensor model, effects of each geometric parameter are checked. For each parameter, a set of five different values is considered with the pre-specified range mentioned in Table 1. Thus 25 initial models are simulated in POD-DMD platform and the variation of the PFD and THD are plotted as shown in Figure 8 and Figure 9. First if the variation of $g_{1}{\;\mathrm{and}\;h}_{i}$ on PFD and THD are analyzed, it is evident from Figure 8a that both $g_{1}{\;\mathrm{and}\;h}_{i}$ have a similar influence on PFD and THD. To analyze their effect in more detail, 2D plots for PFD and THD with the variation of $g_{1}{\;\mathrm{and}\;h}_{i}$ is plotted in Figure 8b. From the 2D plot, the region bounded in between the black and blue dashed lines have the combinations of $g_{1}{\;\mathrm{and}\;h}_{i}$ which fulfills the design requirements of PFD and THD of the proposed sensor module. Thus, $g_{1}{\;\mathrm{and}\;h}_{i}$ can give an important trade off on both PFD and THD.

Now the variations of PFD and THD with other parameters namely, $g_{2},h_{t},\;\mathrm{and}\;w$ are plotted in Figure 8. The effects of $g_{2},h_{t},\;\mathrm{and}\;w$ can be listed as follows:

1. With the increase of $g_{2},$ PFD decreases whereas THD increases. The choice of $g_{2}$ also, depends on the size of the Hall sensor chip placed in between. For an instance, the size of the linear Hall Effect sensor chip (A1324) manufactured by Allegro Microsystem LLC is 1.5 mm. Thus with this limit condition, $g_{2}$ should be chosen as small as possible.
2. The variation of $h_{t}$ does not show very significant effect on the PFD and THD values. The value of t should be selected such that it helps in reduction of the reluctance of the flux path.
3. From Figure 9, an optimal value of 7 mm is perfect for w, which satisfies our design condition for PFD and THD.

Considering the results shown in Figure 7 and Figure 8, a choice for 2D model of the sensor module can be made as $\left(g_{1},g_{2},h_{i},h_{t},w\right)$ = (1.5, 2, 2.8, 3, 7) [mm]. The POD-DMD model simulation of the chosen model shows a PFD value of 0.11T and THD of 1.04%.

#### 4.3.2. Analysis of the Simulation Time in 2D Analysis

To achieve an efficient simulation model, it is important to reduce the simulation time. Therefore, the simulation time to analyze the magnetic sensor module by the tradition FEM and the POD-DMD based reduced model are compared. For a specific set of $g_{1},g_{2},h_{i},h_{t},\;\mathrm{and}\;w$, the 2D FEM model of the sensor module over the stator teeth-slot structure with 5832 nodes, takes around 2.5 s for one simulation. Whereas, the POD-DMD based reduced model with first 10 modes takes 15 ms. Although for a single simulation, the difference in time does not look significant, but for a multi-parameter design analysis and in the optimization process, which requires multiple simulations of the targeted model, the difference in simulation time will significantly large. For an example, a multi-parameter optimization of the sensor module based on Genetic Algorithm (GA) takes parameter values at different generations to find the optimized set of parameters, which results in the analysis of a large set of simulation and the whole process is time-consuming. Thus a significant reduction in time can be achieved if the reduced model is used.

#### 4.3.3. Parametric Modeling for 3D Model

**Figure 10:**
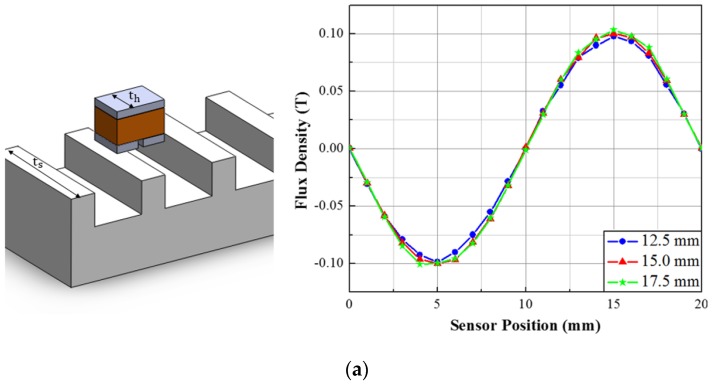

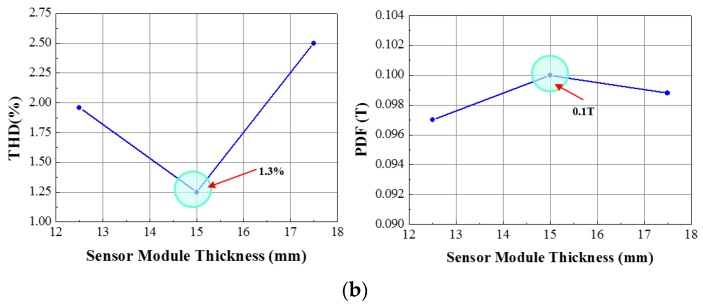
(**a**) 3D configuration of the sensor module and Flux Density variation with thickness; (**b**) Effect of sensor thickness variation on PFD and THD.

The available commercial FE software (FLUX, Ansys) shows fast and promising results in 2D analysis. Thus comparison of the computational time of a simple model using POD-DMD based reduced model with that of the FE model done by commercial FE software will not provide big difference. But if we consider 3D analysis, the commercial 3D software takes a long time to simulate models and the time increases with the increase of the complicacy. Therefore, to check the computational efficiency of the proposed POD-DMD model, we introduced another design parameter for 3D analysis. The sensor thickness $t_{h}$ is considered as shown in Figure 10a and the thickness range is selected in between 10 and 20 mm. Using the POD-DMD reduced model, simulation is carried out with different thickness values. As shown in Figure 10a, the change in thickness does not affect the flux density distribution much. Also to check the variation in PFD and THD in more detail, three values of $t_{h}$, namely, 12.5 mm, 15 mm and 17.5 mm are chosen. 

As presented in Figure 10b, PFD has almost no change with the variation of $t_{h}$ and THD has a minimum at 15 mm with THD of 1.3%. Thus, the thickness is considered as 15 mm as it fulfills our condition of PFD and THD. The system summary and the required time for both 2D and 3D analysis is listed below in Table 2.

#### 4.3.4. Analysis of the Simulation Time in 3D Analysis

The 3D FEM model the magnetic sensor module and the stator teeth-slot structure with 58,735 nodes take around 60 min for one simulation, whereas the time required for the POD-DMD model is 1 min in MATLAB platform with 10 POD modes. Thus, for five different thickness values between 10 and 20 mm, it will take only 5 min to obtain the simulation results. This shows the capability of the reduced model in reducing the computational time.

## 5. Prototype of the Designed Model and Discussion

**Figure 11:**
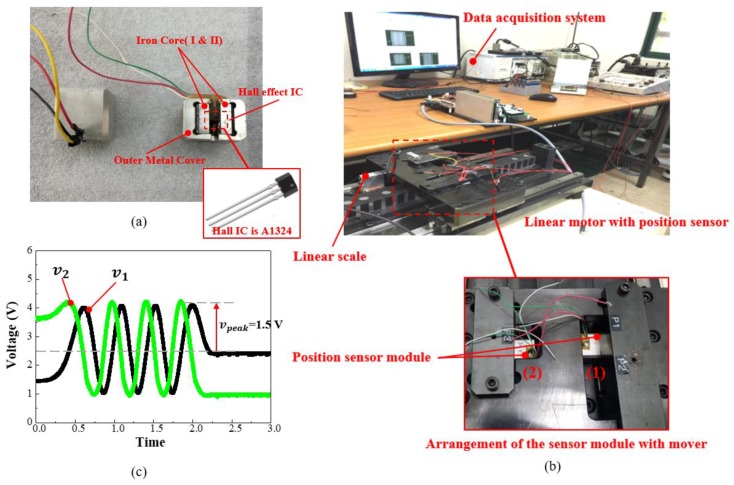
(**a**) Manufactured sensor module; (**b**) Experimental setup with sensor module installed over the linear motor and the data acquisition system; (**c**) Sensor voltage signal.

The structure of the designed prototype with the final design data obtained from the parametric study is shown in Figure 11a. The arrangement of two sensor modules with a phase difference of 90° on the linear motor mover and the data acquisition system is shown in Figure 11b. 

Figure 11c shows the generated outputs by the two sensor modules. From the output data of the manufactured sensor module, following inferences can be made:

1. As shown in Figure 11c, both the signals possess differences in amplitude and offset. The PFD values of the measured signals are also over than 0.1 T. And, the THD values are at 2 and 2.3%, respectively, which is less than the required THD constraint of 3%.
2. The deviation in the PFD and offsets can arise due to a slight deviation in the placement of the Hall IC in between the two cores. While simulation using the PMOR, the PFD value at the center point, in between two iron cores I and II are considered. But in the manufactured prototype, the misalignment of the Hall IC in between iron core I and II, causes deviation in the PFD value.
3. Also, the manufactured sensor module has some mechanical tolerances added to the design parameters during the manufacturing process. This also adds to the deviation in the PFD and THD value of the sensor module. Thus, while selecting the final design parameters, an optimization process considering the manufacturing tolerances should be considered.
4. This gives a scope to analyze the sensor module with further research work based on the reliability design optimization techniques.

**Figure 12:**
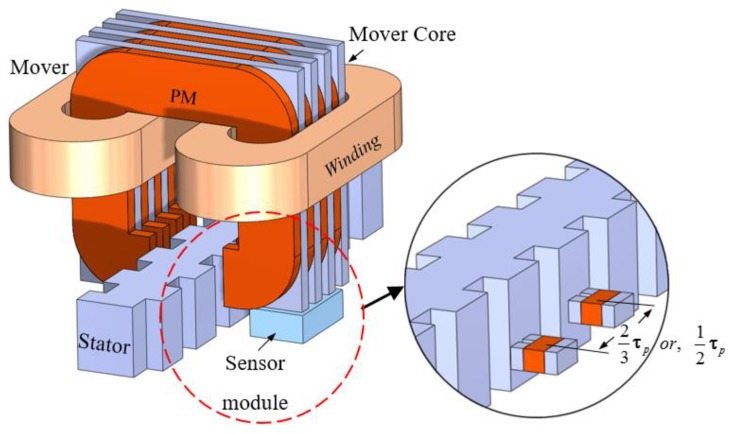
Arrangements of the sensor modules with the mover over the stator to detect the moving position of the mover.

**Figure 13:**
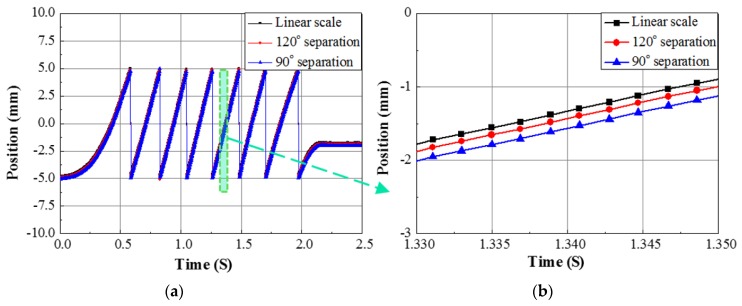
(**a**) Mover position of the linear motor detected using the sensor module and the comparison of the position data with that of the linear scale; (**b**) Deviation of the sensor module data with that of the linear scale [7].

Finally, as shown in Figure 12, two different arrangements with separation of 90° or 120° electrical between two sensor modules over the stator tooth-slot structure is considered to detect the moving position of the mover unit of the linear motor [7]. During the position analysis, the amplitude of the sensor output voltage signals is adjusted to unit amplitude to minimize the amplitude differences. For 90° separations, the position value is obtained by applying arctangent on the output values of the sensors. For 120° separations, three to two phase transformation is done to reduce the three multiples of harmonic components. The final output thus obtained is converted to position data by the utilizing of arctangent function. The position waveforms are shown in Figure 13a. The position information obtained using the 120° mode has a standard deviation of 0.10 mm from the reference linear scale signal, whereas the 90° mode position signal shows a deviation of 0.23 mm from the reference [7]. Figure 13b shows the zoom in waveforms within the broken line portion of Figure 13a. 

A more detailed analysis on the comparison of the two abovementioned position detection methods can be found in our previously published work [7]. Moreover, as shown above, the deviation arises due to the errors associated with the manufacturing process. This error can be eliminated by considering the effect of the tolerance into the design process.

## 6. Conclusions

A new computationally efficient design approach for the magnetic sensor module using the model order reduction (MOR) method is proposed in this paper. A mathematical framework for the MOR is designed using the proper orthogonal decomposition (POD) and dynamic mode decomposition (DMD) method. Firstly, the mathematical framework is tested on a benchmark equation to check the validity of the designed method compared with the previously developed Discrete Empirical Interpolation Method (DEIM). Considering the capability of the proposed method in approximating a full model, it is implemented in designing and parametric study of a magnetic position sensor for linear motor mover position detection. With the final parameters obtained using the proposed method, a sensor prototype is designed and is tested for the required Peak Flux Density (PFD) and Total Harmonic Distortion (THD). The manufactured sensor module output data fulfills the desired constraint conditions of PFD greater than 0.1 T and THD less than 3%, respectively. The sensor prototypes are used to detect the mover position on two different modes, 90° and 120° modes respectively. The position information thus obtained are compared with that of the linear scale data. The position data obtained using the sensor prototype contains a small deviation from the linear scale data. The deviation in the position data arises due to the manufacturing tolerance incur during the manufacturing process. To improve the sensor output, a further study on reliability based design optimization considering manufacturing tolerances will be considered in the future research.

## Figures and Tables

**Table 1:** Specification for the Stator and Sensor module.

Stator	Values (mm)	Sensor Module	Range (mm)
Tooth width, $W_{t}$	7	$g_{1}$	1~3
Slot width, $W_{s}$	13	$g_{2}$	2~4
Tooth Height, $t_{h}$	5	$h_{i}$	0~3
Pole pitch, $\tau_{p}$	10	h_t_	5~9
		w	3~5

**Table 2:** Analysis summary and timing.

Method	2D FEM	2D PMOR	3D FEM	3D PMOR
Snapshot Size	5832 × 100	5832 × 10	58,735 × 100	58,735 × 10
Time	2.5 s	15 ms	60 m	1 m
Software used	Cedrat Flux	Matlab	Cedrat Flux	Matlab
